# Evaluation of basil (*Ocimum basilicum*) accessions under different drought conditions based on yield and physio-biochemical traits

**DOI:** 10.1186/s12870-023-04554-8

**Published:** 2023-10-28

**Authors:** Mehdi Rahimi, Mojtaba Mortazavi, Ameneh Mianabadi, Sandip Debnath

**Affiliations:** 1 Department of Biotechnology, Institute of Science and High Technology and Environmental Sciences, Graduate University of Advanced Technology, Kerman, Iran; 2 Department of Ecology, Institute of Science and High Technology and Environmental Sciences, Graduate University of Advanced Technology, Kerman, Iran; 3 Department of Genetics and Plant Breeding, PalliSiksha Bhavana (Institute of Agriculture), Visva-Bharati University, Sriniketan, West Bengal 731236 India

**Keywords:** Water deficit conditions, Mean comparison, Multivariate analysis, Proline content

## Abstract

**Background:**

Basil is one of the most famous herbs, which has broad usage as a fresh vegetable and therapeutic and pharmaceutical services. The main abiotic stress limiting basil production globally is drought. As a result, appropriate drought screening-which effectively separates high-yielding but drought-sensitive genotypes from drought-tolerant genotypes-is necessary for the optimal selection of high-yielding basil cultivars under drought stress conditions. So, a split plot experiment with three replications based on a completely randomized design were carried out in a pot under field conditions for this investigation. Water levels (full irrigation or control, moderate stress, and severe stress) were assigned as main plots, while 22 basil accessions were given as sub-plots. In this study, leaf yield as well as physio-biochemical traits had measured on accessions.

**Results:**

Our results revealed large variation in yield, essential oil (%), protein, proline, chlorophyll, total phenol and flavonoids traits across the 22 accessions. The percentage of leaf yield reduction in moderate drought stress than normal conditions showed that G1 (−6.5%), G17 (−7.05%), G20 (−9.01%), and G12 (−10.9%) accessions had the least changes, respectively. Although in severe drought stress than normal conditions, the G1 (−32.01%), G12 (−33.12%), G4 (−33.24%), G7 (−34.11%), and G17 (−34.93%) accessions had the least amount of change in plant leaf yield, respectively. Furthermore, the highest yield reduction occurred in moderate and severe stress conditions in G18 (−25.36%) and G8 (−42.98%) accessions, respectively. Cluster analysis based on the ward method in both conditions (moderate and severe drought conditions) placed the accessions in three groups, and accessions were identified as tolerant, whose average traits in that group were higher than the total average. The principal component analysis also showed that in moderate drought conditions, the first two components explained about 95.28% of the total variation, while in severe drought conditions, these two components explained about 96.37% of the total variation.

**Conclusions:**

The different multivariate analyses (cluster analysis, PCA, mean comparison) were used to identify tolerant and sensitive accessions based on all traits. The accessions G3, G4, G6, and G7 were found to be tolerant to stress, while G10, G15, G16, and G20 were found to be sensitive to drought. These accessions are a useful step in producing drought-tolerant, high-yielding accessions and can be utilized in breeding programs for basil.

## Background

Basil is a medicinal plant and an important vegetable of the mint family. Basil is an herbaceous and annual plant that has great diversity in terms of morphology and secondary compounds, especially for essential oil content [1, 2]. This genus has 50 to 150 or even more herbaceous and shrub species. The *Ocimum* genus includes several species such as *Ocimum gratissimum*, *Ocimum carnosum*, *Ocimum sanctum*, *Ocimum canum*, and *Ocimum basilicum*, and the *Ocimum basilicum* species has more than 60 varieties [3, 4]. For this reason, it is considered one of the most important genera in the mint family [5], among which *Ocimum basilicum* is the most important economic species. Basil is a long-day and cold-sensitive plant that grows well in moist soil with proper drainage, in full sunlight, and in warm weather. Basil grows naturally in tropical and subtropical regions and is mostly found in Asia, Africa, Central and South America [1]. Basil has been mentioned as an important medicinal plant in most pharmacopoeias. Its leaves and essential oil are used to treat some ailments, such as headache, cold, as well as severe diseases like diarrhea and kidney failure [6]. The active ingredients of this plant are used to treat flatulence, strengthen the digestive system, relieve pain and fatigue [7, 8]. Basil is also an edible vegetable whose dried leaves are added to many foods as a flavouring agent and to aid digestion, and it has long been used to protect food from spoilage and also as a fresh herb [9]. Flavonoids, phenolic acids, and essential oils are only a few of the active substances found in basil. Eucalyptol, linalool, and methyl chavicol are the three main essential oils present in basil. Basil also contains phenolic acids including caffeic acid and rosmarinic acid, as well as flavonoids such as vicenin, orientin, and apigenin. These substances give basil its distinctive scent and therapeutic actions, including anti-inflammatory, antioxidant, and antibacterial activities. Depending on the cultivar, environmental circumstances, and time of harvest of the basil plant, these active compounds’ precise concentration and composition may change [10, 11].

Drought is a natural phenomenon that is typically defined as a period without considerable rainfall (lack of water), resulting in extensive crop damage and a massive loss of harvests [12]. Drought-caused water scarcity is a big problem in Iran. Iran is just one of several countries that have had to confront the growing problem of depleting water resources as a result of rising populations and the consequent demand for food and water. Iran, like many other developing countries, has a looming water crisis, and the future of its food supply is tied directly to the reliability of its water infrastructure and supply [13, 14]. So, it is important to work on a plan to mass-produce high-quality basil genotypes that can thrive even in drought conditions [15]. The arid and semiarid regions of Iran receive an average of 240 millimetres of rainfall each year [16]. One of the most critical stresses for crop plants is a lack of water. But water constraints aren’t unique to arid regions; in fact, uneven rainfall in some areas can make it tough for plant growth. Loss of yield is common when a plant is subjected to this kind of stress [17]. Yield loss due to drought is caused by its impact on every part of the plant system. Crop damage from water deficits during certain phases of plant development is greater than during other stages [18, 19]. Thus,variations in response to water stress can be observed in morphology, physiology, and molecular responses [20, 21]. Therefore, breeding efforts should take into account yield and its components as one of the most essential criteria for the selection of suitable or tolerant plants [18, 19]. Thus, the ability to resist or tolerate drought is considered a desirable physiological and breeding trait.

According to a drought research conducted on basil, under moderate and severe water stress, drought stress decreased dry matter yields by 15% and 28%, respectively. Moreover, the results showed that the highest amount of proline and chlorophyll was in severe and normal stress conditions, respectively [22]. In the study of Kalamartzis, Papakaloudis [23], twenty landraces of basil were studied under different drought stress conditions for three years. Over the course of the three years, the data showed a 20% loss in dry matter production and a 21% reduction in essential oil yield in all genotypes evaluated, with the lowest irrigation treatment showing the greatest reductions in comparison to the control treatment [23]. Under drought stress, the effects of mycorrhiza and plant growth boosters were studied on basil. Under extreme stress, the data showed a rise in proline and a fall in chlorophyll and shoot dry weight. Furthermore, the outcomes demonstrated that basil’s resistance to drought stress was raised by the application of mycorrhiza and plant growth stimulants [24].

Previous research has demonstrated the complex and multigenic nature of plant responses to environmental stress, and the roles of many activated genes are still not fully understood. This complexity makes selecting and breeding drought-tolerant cultivars extremely challenging. In order to mitigate the consequences of environmental stress, plants have evolved excellent defense mechanisms by modifying their tolerance potential through integrated molecular and cellular responses. As a result, performing an appropriate drought screening that clearly separates high-yielding but drought-sensitive genotypes from drought-tolerant genotypes is necessary for the optimal selection of high-yielding basil cultivars under drought stress conditions [25].

Thus, the aim of the present study was to characterise the basil accessions under drought stress conditions and to select the best drought-tolerant accessions for the future drought tolerance breeding program based on evaluation yield and physio-biochemical traits.

## Materials and methods

### Plant material

This study conformed with all applicable Iranian institutional, national, and international regulations. As the study’s plant material, landraces, ancient, and modern accessions of basil from various geographic regions were purchased and gathered from various businesses; no particular permits were needed to acquire the plant materials. Dr. Mahmood Maleki identified the species, and voucher specimens (numbers 201 to 222) were deposited in the Graduate University of Advanced Technology Herbarium to be used for botanical research upon formal request (Table 1).

**Table 1:** The name and characteristics of basil accessions

Voucher	Code	Landrace/cultivar	Origin
201	G1	Italian Genovese	Italy
202	G2	Particolored	Iran
203	G3	Red Rubin	Denmark
204	G4	Lemon	India
205	G5	Afghan Green	Afghanistan
206	G6	Lettuce	Japan
207	G7	Purple	Iran
208	G8	Turkish Arzuman (Yesil Feslegen)	Turkey
209	G9	Cinnamon	Mexico
210	G10	Persian Green	Iran
211	G11	Napoletano	Italy
212	G12	Flower pesto	Italy
213	G13	Midnight	Africa
214	G14	Dark opal	USA
215	G15	Horapa	Thailand
216	G16	Green Mobarakeh	Iran
217	G17	Purple crab	Italy
218	G18	Italian Violetto	Italy
219	G19	Holy Thai	Thailand
220	G20	Black	Turkey
221	G21	Italian Green	Italy
222	G22	Holy (Tulsi)	India

### Experimental site

This experiment was carried out in the year of 2021 at the Graduate University of Advanced Technology and is located in Mahan city at a latitude of 30° 3′ 30.06″ north and a longitude of 57° 17′ 37.93″ east, at an elevation of 2020 m above mean sea level.

### Pot preparation and soil of the experiment

The plastic pots 19 cm in diameter, 15 cm in height, and having a capacity of 3 kg of soil were used in this experiment. In addition, the soil of the pots was a combination of agricultural soil, sand, and animal manure in a ratio of 2:2:1. The physical and chemical characteristics of the pot soil are listed in Table 2. 25 seeds were planted in each pot, and after germination and establishment of seedlings, five plants were kept in each pot and the rest were thinned.

**Table 2:** Some physical and chemical characteristics of soil

C (%)	EC (dS/m)	Soil texture (%)	K (ppm)	N (%)	pH
2.78	2	Sandy–clay–loam	353.75	0.23	7

### Experimental design and drought treatment

Since there haven’t been any studies screening basil accessions for drought tolerance, 22 basil accessions (Table 1) were examined in this study using a replicated design. The experiment was conducted in the form of a split plot based on a completely randomized design (CRD) with three replications in pots under field conditions in 2022. Drought stress was applied as the main plot in three levels: normal (irrigation cycle of five days), moderate (irrigation cycle of nine days), and severe (irrigation cycle of 13 days) stress conditions. Accessions were also considered sub-plot factors at 22 levels. Irrigation treatment was applied at three levels of normal irrigation, medium and severe water deficit stress, respectively, with the field capacity of 85, 60, and 40% of usable moisture. Previously, this amount of field capacity was measured based on the test site conditions and was used as the irrigation cycle.

### Characteristics studied and plant sampling

The observations for leaf yield as well as biochemical and physiological traits including essential oil (%), protein, proline, soluble sugars, chlorophyll a, b, total chlorophyll, carotenoids, total phenol and total flavonoids traits were recorded on plants for each genotype in each replication in each environment, and the average of them as per plant was used as the final data for statistical analysis. The basil plants’ leaves were picked, placed in foil in liquid nitrogen, and transferred to the laboratory for physiological and biochemical characteristics measurement using the techniques outlined by Sudhakar, Latha [26].

### Leaf yield

The weight of the fresh leaves of each plant was measured on a digital scale at the maturity stage.

### Chlorophyll content

Chlorophyll a and b concentrations as well as total chlorophyll were measured using the Arnon’s method [27]. A test tube containing 0.2 g of fresh leaf was ground with 10 mL of 80% acetone, and the absorbance was measured using a spectrophotometer at 663 and 645 nm. Using the following formulae as provided by by Sudhakar, Latha [26]. To ascertain the quantity of chlorophyll within the extract, it is imperative to measurably comprehend its concentration denoted as milligrams of chlorophyll per gram of tissue.

1 $$\begin{aligned}mg\,chlorophyll\,a/g\,tissue & =[\left(12.7\times A663\right)\\ & \quad -\left(2.69\times A645\right)]\times \frac{v}{1000\times w} \end{aligned}$$

2 $$\begin{aligned}mg\,chlorophyll\,b/g\,tissue& =[\left(22.9\times A645\right)\\ & \quad -\left(4.68\times A663\right)]\times \frac{v}{1000\times w}\end{aligned}$$

3 $$\begin{aligned} mg\,total\,chlorophyll/g\,tissue & =\left[\left(20.2\times A645\right)\right.\\ & \quad \left. -\left(8.02\times A663\right)\right]\times \frac{v}{1000\times w}\end{aligned}$$

Where: W = fresh weight of tissue extracted, V = final volume of chlorophyll extract, and A = absorbance at particular wavelengths.

### Total carotenoids

The method developed by Price and Hendry [28] was used to measure the total carotenoids. This method involved utilizing the sample extract that was obtained using the acetone method to determine chlorophyll. According to Sudhakar, Latha [26], the pigment content is determined in mg/g of fresh weight and the absorbances are measured at 663, 645, and 480 nm in a spectrophotometer using the following formula:

4 $$\begin{aligned}mg\,total\,carotenoids/g\,tissue& =[A480+\left(0.114\times A663\right)\\ & \quad -\left(0.638-A645\right)]\times \frac{v}{1000}\times w\end{aligned}$$

### Proline

To determine the proline content of leaves, we done the Bates et al. technique [29]. The extract was centrifuged to obtain the supernatant, which was then mixed with 2 ml of ninhydrin reagent and 2 ml of pure acetic acid. The resulting mixture was heated at 100 °C in an air bath for one hour. The tubes containing the liquid were placed in the ice bath as soon as possible. After adding 4 ml of toluene to the mixture, the tubes underwent thorough vortexing. For 15 to 20 min, the tubes were stacked to form two different layers. The upper colour phase, which contained toluene and proline, was used to calculate the proline concentration. The proline concentration was calculated using a standard curve after the absorbance at 518 nm was obtained.

### Protein

In cold water with phosphate buffer, the proteins were isolated from the aerial portions between 0 and 4 °C. After that, a consistent blue colour was achieved using Coomassie Brilliant Blue (CBB) G-250. A spectrophotometer reading the absorbance at 595 nm was made after 25 min. Protein concentrations were calculated using Bradford’s method [30].

### Reducing sugar

0.02 g of the plant’s leaves were combined with 10 ml of distilled water in a Chinese mortar and boiled on an electric stove. Once the solution reached boiling point, the heat was turned off and the solution was filtered using filter paper. Test tubes were then filled with 2 ml of the resulting extract and 2 ml of a copper sulphate solution. The tubes were securely closed with cotton and heated in a hot bath at 100 °C for 20 min. After cooling, 2 ml of the phosphomolybdic acid solution were added to the tubes, resulting in the appearance of a blue tint. The color was then evenly distributed by shaking the test tubes erratically with a vortex device. Using a spectrophotometer, the Somogyi method [31] was used to measure the absorbance intensity of the solutions at 600 nm wavelength in order to demonstrate the concentration of reducing sugar using the standard curve.

### Total phenolic content

The total phenolic content of the extract was ascertained using the Folin-Ciocalteu method [32]. 200 ml of a herbal extract were mixed with 0.8 ml of sodium carbonate (7.5%), and 1 ml of the Folin-Ciocalteu reagent. All samples’ absorbance was assessed with a spectrophotometer at 750 nm after 1.5 h of dark storage at 30 °C. The amount of phenol in the test sample was calculated using the standard curve and represented as mg phenols/100 g of material.

### Essential oil

Using equipment of the Clevenger type that was based on the European Pharmacopoeia, the percentage of essential oil was also calculated [33]. After drying the essential oil’s water with sodium sulphate to remove impurities, the dry weight of the essential oil was estimated, along with its quantity and essential oil content.

### Total flavonoid content

The total flavonoid content of the dense extract was determined by the colorimetric method [34] based on the standard curve of the standard solution of (+)-catechin at 0, 20, 40, 60, 80, and 100 mg/l. 4 ml of distilled deionized water (dd H_2_O) and an aliquot of the extracts (1 ml) were added to a10 ml volumetric flask. Then, 0.3 cc of 5% NaNO_2_ was added. 0.3 ml of 10% AlCl_3_ was added after 5 min. At the six-minute mark, 2 ml of NaOH 1 M were added, and dd H_2_O was used to bring the total volume to 10 ml. A spectrophotometer was used to measure the solution’s absorbance at a wavelength of 510 nm, and the results were represented as milligrams of catechin equivalents (CE) per 100 g of dry weight (mg CE/100 g dw).

### Statistical analysis

The split plot design based on CRD design was utilised for the variance analysis of the data using SAS software version 9.4 [35], and Duncan’s test was applied at the 1% probability level to compare averages. The biochemical and physiological trait datasets were subjected to principal component analysis (PCA) using PAST 4.0.3 software [36], and hierarchical clustering using the UPGMA method and Euclidean distance measurement were also carried out on the datasets. Using the factoextra package and the R programming language, the number of groups was calculated [37].

## Results

### Descriptive analysis

In the present investigation, 22 basil entries were analysed for important physio-biochemical parameters under three different moisture conditions. At 60% field capacity (moderate drought condition), the reduction in the physio-biochemical traits ranged from − 23.43 to 59.43%. The summary statistics of the accessions under different levels of drought stress revealed that chlorophyll b, total chlorophyll, and chlorophyll a were reduced by 33.05%, 28.71, and 26.82% in the severe drought condition, while they were reduced to 17.94%, 21.77%, and 23.43% in the moderate drought compared to the control condition (Table 3), respectively. Some traits increased under stress conditions instead of decreasing compared to normal conditions. These traits included protein, proline, sugars, total phenol, and total flavonoids traits.

**Table 3:** Descriptive statistics for studied traits in different stress conditions

Discriptive index	Env.	Traits										
		Leaf yield	Essential oil (%)	Protein	Proline	Soluble sugars	Chl. a	Chl. a	Total Chl.	Carotenoids	Total phenol	Total flavonoids
Min	S1	7.64	0.51	0.46	0.06	0.77	7.78	2.39	10.25	2.58	32.46	3.14
	S2	6.57	0.38	0.68	0.19	0.96	5.92	2.31	8.23	2.24	36.11	4.40
	S3	4.87	0.53	0.87	0.20	1.07	5.36	1.80	7.16	2.08	42.95	5.50
Max	S1	15.50	2.48	0.81	0.61	1.14	14.34	7.19	21.53	3.50	60.41	8.15
	S2	13.10	2.02	1.14	0.83	1.37	12.34	5.81	18.14	3.08	67.30	9.32
	S3	10.35	1.91	1.44	1.24	1.58	10.53	4.63	15.15	2.91	71.73	10.69
Mean	S1	11.79	1.37	0.62	0.32	0.91	11.50	5.00	16.50	3.09	43.81	5.58
	S2	9.81	1.30	0.92	0.51	1.16	8.81	4.10	12.91	2.72	47.16	7.26
	S3	7.28	1.13	1.15	0.73	1.36	8.42	3.35	11.76	2.50	56.82	8.25
Stand. dev	S1	2.52	0.65	0.12	0.18	0.11	2.01	1.63	3.61	0.28	9.56	1.75
	S2	2.13	0.47	0.13	0.22	0.12	2.01	1.29	3.28	0.28	9.30	1.57
	S3	1.65	0.36	0.19	0.36	0.16	1.46	0.77	2.22	0.25	9.09	1.67
Coeff. var	S1	21.35	47.21	18.86	55.64	12.56	17.44	32.52	21.87	9.18	21.82	31.35
	S2	21.66	36.46	14.10	43.94	10.60	22.84	31.51	25.38	10.19	19.73	21.68
	S3	22.67	32.02	16.63	48.69	11.53	17.36	22.89	18.87	9.96	15.99	20.24
% Change than normal condition	S2	−16.73	−5.24	46.74	59.43	27.85	−23.43	−17.94	−21.77	−11.76	7.65	30.08
	S3	−38.19	−17.61	84.19	130.82	50.24	−26.82	−33.05	−28.71	−19.01	29.7	47.71

S1, S2 and S3 are Normal condition, Moderate drought condition and Severe drought condition, respectively

The C.V. % in (Table 3) varied from 9.18% (carotenoids) to 55.64% (proline) for the traits in normal condition. Proline and essential oil (%) had the highest percentages of phenotypic variation under normal conditions, at 55.64% and 47.21%, respectively (Table 3). Moreover, the C.V. % of the leaf yield was about 21.35%. The higher values of the coefficient of variation indicate that a response to selection can be expected for their improvement. The C.V. % of moderate drought stress varied between “10.19 and 48.69’’ for the studied traits (Table 3). The proline showed the highest value of C.V.% and the lowest value was observed for carotenoids. The amount of C.V. % observed for essential oil and leaf yield in these conditions was equal to 34.46% and 21.66%, respectively (Table 3). Table 3 showed that the value of C.V. % of the studied traits varied between 9.96 and 48.69 under severe drought conditions, and the traits carotenoids and proline had the lowest and highest values, respectively. In this condition, the traits under study showed good diversity, and traits such as leaf yield and essential oil had 22.67% and 32.02% of variation, respectively.

### Variance analysis and mean comparison

The ANOVA has indicated significant variation among the genoytpes as well as accession× environment intraction (Table 4) which indicates that significant differences exist among accessions and the expression has shown a non-linear relation with environments. As the genotype×environment component was significant for all traits, the comparison of the mean values of a trait of each genotype in each environment was taken into consideration and presented in (Table 5). The accessions G1, G3, and G5 had the highest values, while G19 and G22 had the lowest values for protein, proline, and sugar contents under severe drought conditions. As higher protein, proline, and sugar content confer higher tolerance to stress, G1, G3, and G5 are better suited for drought stress. The accessions G4, G3, and G7 had the highest values for leaf yield under normal drought conditions, indicating their suitability for normal conditions, while the highest yield was recorded in moderate and severe stress conditions for the G4 and G7 accessions (Table 5).

**Table 4:** Split plot variance analysis of the biochemical traits in basil accessions in different stress environments based on completely random design with three replications

	Mean square of traits					CV%
S.O.V	Environments	Reps within Environment	Genotype	Environments×Genotype	Error	
DF	2	6	21	42	126	
Leaf yield	336.10^**^	6.42	38.74^**^	0.99^**^	0.037	1.99
Essential oil (%)	1.019^**^	0.0429	0.599^**^	0.867^**^	0.00028	1.32
Protein	4.581^**^	0.025	0.190^**^	0.0056^**^	0.00025	1.77
Proline	2.865^**^	0.0239	0.568^**^	0.0291^**^	0.00014	2.28
Soluble sugars	3.437^**^	0.0241	0.0371^**^	0.0605^**^	0.000061	0.69
Chl. a	186.211^**^	2.630	29.019^**^	0.7936^**^	0.0074	0.89
Chl. b	45.149^**^	1.894	13.353^**^	0.6744^**^	0.00598	1.86
Total Chl.	403.119^**^	8.981	81.35^**^	2.3403^**^	0.01346	0.85
Carotenoids	5.787^*^	1.084	0.2098^**^	0.2242^**^	0.004064	2.30
Total phenol	3012.732^**^	2.528	769.418^**^	6.0002^**^	0.00488	0.14
Total flavonoids	119.729^**^	0.421	7.420^**^	8.783^**^	0.00165	0.58

^*^ and ^**^ are significant at 5% and 1% probability level, respectively

**Table 5:** Mean comparison of the studied traits in normal and drought stress conditions

	genotype	Traits										
		Leaf yield	Essential oil (%)	Protein	Proline	Soluble sugars	Chl. a	Chl. b	Total Chl.	Carotenoids	Total phenol	Total flavonoids
S1	G1	10.51p-r	2.072c	0.807s-u	0.609lm	0.983b1c1	12.25e	5.37g-i	17.63h	3.186b-e	34.43q1	5.79a1
S2	G1	9.82s-w	0.791v	1.138fg	0.830ef	1.323hi	8.95st	4.32pq	13.27t	2.393w-c1	38.56l1m1	7.201x
S3	G1	7.15f1h1	1.546h	1.44a	1.242a	1.576a	8.96st	3.38u-w	12.34w-y	2.499t-z	49.71w	9.51f
S1	G2	13.91bc	1.025st	0.802s-u	0.580mn	0.767m1	13.01c	6.61cd	19.62de	3.504a	33.79r1	4.88e1
S2	G2	11.89h-k	1.319no	1.092gh	0.805fg	1.023y-a1	9.83no	5.31h-j	15.14n	2.718n-t	37.23n1	7.35vw
S3	G2	8.63y-a1	1.187q	1.413ab	1.167b	1.438e	9.24q-s	3.95rs	13.20tu	2.763l-r	48.27yz	7.92s-u
S1	G3	15.4a	1.077rs	0.782t-w	0.556no	0.97c1d1	13.61b	6.87bc	20.49c	3.118b-h	58.93l	7.96r-t
S2	G3	12.24g-i	1.799f	1.048hi	0.790f-h	1.129st	11.87f	5.62e-g	17.50h	3.084c-i	58.53m	5.74a1
S3	G3	9.69t-w	1.017t	1.392ab	1.162b	1.567a	10.31l	4.41op	14.72o-q	2.248a1-e1	70.66c	8.51no
S1	G4	15.5a	1.777f	0.778t-w	0.541n-p	0.872e1f1	14.34a	7.18a	21.52a	3.496a	60.41j	4.31g1
S2	G4	13.09d-f	0.381b1	1.047hi	0.771gh	1.205op	12.33de	5.81e	18.14g	2.438v-b1	67.301e	5.48b1-d1
S3	G4	10.34q-t	1.493h-j	1.373b	1.142bc	1.073v-x	10.52l	4.62no	15.15n	2.361y-d1	71.73a	5.5b1c1
S1	G5	14.45b	2.010d	0.744v-y	0.513p-r	0.781l1m1	11.33i-k	4.72mn	16.06lm	3.133b-g	45.97b1	6.52z
S2	G5	11.7i-l	1.152q	1.041hi	0.764h	1.021za1	7.85y-b1	3.18v-y	11.03b1c1	2.763l-r	50.56v	4.39g1
S3	G5	9.21w-y	0.793v	1.377b	1.142c	1.491cd	7.67a1-c1	3.02yz	10.69c1d1	2.175c1-e1	61.39i	9.79e
S1	G6	15.17a	2.479a	0.728w-z	0.477rs	0.802i1-l1	11.51g-i	4.84l-n	16.34kl	2.661o-u	58.06n	5.36c1d1
S2	G6	11.93h-j	0.802v	1.023ij	0.721i	1.093u-w	8.01x-z	3.99rs	12.01yz	2.288z-e1	58.32m	8.92h
S3	G6	8.92x-z	1.285op	1.307cd	1.112c	1.291jk	8.16wx	3.16v-y	11.33a1b1	2.458u-a1	69.86d	9.97cd
S1	G7	15.23a	0.596xy	0.695y-b1	0.433tu	0.856f1g1	13.91b	7.13ab	21.04b	3.262bc	59.24k	4.62f1
S2	G7	12.47f-h	0.906u	0.999i-l	0.662jk	1.033yz	11.75fg	5.64e-g	17.39hi	3.018d-j	64.21g	8.17p
S3	G7	10.03r-v	1.287op	1.312c	1.112c	1.307ij	10.38l	4.375	14.75n-p	2.617p-v	71.28b	8.18p
S1	G8	13.34c-e	2.008d	0.684z-b1	0.425t-v	8.9	13.03c	6.49d	19.52e	2.784k-q	42.22h1	6.5z
S2	G8	11.26k-n	1.914e	1.009i-k	0.631kl	1.195op	10.26lm	5.43f-h	15.69m	2.974e-l	45.64c1	8.47no
S3	G8	7.61c1f1	0.556yza1	1.276cd	1.051d	1.409f	9.35qr	3.91rs	13.27t	2.700n-t	52.53s	8.66k-m
S1	G9	7.63c1-f1	0.651wx	0.667a1-c1	0.383w-y	0.954d1	7.77z-c1	2.47b1c1	10.25e1f1	3.319ab	32.45u1	3.71i1
S2	G9	6.59h1i1	0.665w	0.981j-l	0.620lm	1.369g	5.92i1	2.31c1d1	8.23j1	2.591q-x	36.11o1	8.01q-s
S3	G9	4.87m1	1.087r	1.253d	0.862e	1.293jk	5.36j1	1.80e1	7.16k1	2.662o-u	42.95f1	10.28b
S1	G10	12.6fg	0.573yz	0.664b1c1	0.345y-a1	1.049xy	12.89c	5.68ef	18.57f	2.847j-o	33.96r1	4.67f1
S2	G10	10.62n-r	1.661g	0.955k-o	0.601lm	1.232mn	9.51pq	5.12i-k	14.64pq	2.505s-z	38.43m1	8.53m-o
S3	G10	7.4e1-g1	0.678w	1.195e	0.863e	1.181pq	9.01st	3.78st	12.80uv	2.367y-d1	48.73x	5.54b1
S1	G11	10.14q-u	0.793v	0.621c1d1	0.339za1	1.038yz	9.78n-p	3.41uv	13.19tu	3.120b-h	48.18z	8.09p-r
S2	G11	8.19a1-c1	1.657g	0.961k-n	0.508p-r	1.024y-a1	71.3	3.02yz	10.15e1f1	3.025d-j	52.98r	8.81h-j
S3	G11	5.92j1k1	1.37mn	1.149ef	0.673j	1.536b	7.53d1	2.77za1	10.31d1-f1	2.371x-d1	63.39h	8.59l-n
S1	G12	11.22l-o	1.251p	0.614c1d1	0.268b1	0.842g1h1	11.95f	4.78mn	16.73jk	3.299a-c	42.43g1h1	3.56j1k1
S2	G12	9.9r-v	1.372mn	0.914n-p	0.497q-s	1.348gh	8.25wx	4.02rs	12.28w-y	2.918g-n	46.03b1	9.31g
S3	G12	7.5d1g1	0.694w	1.105fg	0.629kl	1.284jk	8.84tu	3.32u-x	12.17xy	2.906h-n	54.84q	8.86hi
S1	G13	13.54cd	1.335no	0.596	0.230b1-d1	1.133r-t	12.57d	5.71e	18.28fg	3.232b-d	45.51c1	8.15pq
S2	G13	11.316	0.827v	0.888pq	0.390v-x	1.256lm	9.38qr	4.95k-m	14.34qr	2.612p-w	48.76x	5.45b1-d1
S3	G13	7.86b1-e1	1.450kl	1.093gh	0.581mn	1.094uv	9.08r-t	3.53tu	12.62vw	2.162d1e1	59.44k	10.06c
S1	G14	8.38z-b1	2.035cd	0.584d1e1	0.210d1-f1	0.961c1d1	8.42vw	2.65a1b1	11.07b1c1	3.252bc	33.23s1	6.73y
S2	G14	6.65h1i1	1.465k	0.856q-s	0.390v-x	1.146rs	6.46g1h1	2.38b1-d1	8.85h1i1	2.235b1-e1	37.27n1	4.59f1
S3	G14	5.23l1m1	0.897u	1.032ij	0.543n-p	1.211no	6.38g1h1	2.42b1-d1	8.81h1i1	2.387x-c1	45.62c1	5.74a1
S1	G15	10.6o-r	2.286b	0.55e1f1	0.198d1-f1	0.824h1i1	13.11c	6.84c	19.95d	2.956f-l	36.03o1	3.85h1
S2	G15	8.46z-b1	1.623g	0.856q-s	0.385v-y	1.067wx	11.06k	5.59e-g	16.65jk	2.734m-r	39.81k1	6.46z
S3	G15	6.24i1-k1	0.773v	1.044hi	0.528o-q	1.269kl	9.71op	4.10qr	13.81s	2.545r-y	50.82u	9.86de
S1	G16	10.44p-s	0.505a1	0.527f1g1	0.176f1-h1	1.135r-t	11.16jk	3.91rs	15.08no	3.236b-d	32.94t1	7.43v
S2	G16	8.46z-b1	1.309o	0.827r-t	0.392u-x	1.086vw	7.51d1	3.18v-y	10.70c1d1	2.948f-m	36.25o1	5.35d1
S3	G16	6.17i1-k1	1.067rs	1.04hi	0.459st	1.158qr	7.56b1-d1	2.77za1	10.33d1-f1	2.724n-s	44.97d1	5.56b1
S1	G17	11.09l-p	1.394lm	0.501f1-h1	0.154g1-i1	0.788k1-m1	13.14c	6.87bc	20.01d	3.501a	42.59g1	3.14l1
S2	G17	10.31q-t	1.897e	0.821r-t	0.339za1	1.213no	11.66f-h	5.61e-g	17.28hi	2.237b1-e1	47.24a1	8.07p-r
S3	G17	7.21e1h1	1.419k-m	0.977j-m	0.409u-w	1.474d	10.01mn	4.16p-r	14.16rs	2.866i-o	56.53o	8.03q-s
S1	G18	9.64u-w	0.907u	0.502f1-h1	0.147h1i1	0.878e1f1	9st	3.14w-y	12.14xy	2.717n-t	55.78p	3.47k1
S2	G18	7.19f1h1	1.486ij	0.791t-v	0.254bic1	1.285jk	6.77f1	2.56a1-c1	9.33g1	2.663o-u	54.68q	8.75i-k
S3	G18	5.72k1l1	1.908e	0.947l-o	0.367x-a1	1.424ef	7.35d1e1	2.75a1	10.11f1	2.906h-n	64.61f	7.15x
S1	G19	9.23w-y	0.568yz	0.486g1h1	0.136h1-j1	0.815h1-j1	8.61uv	3.06xy	11.68za1	2.575q-y	4.41e1	4.69f1
S2	G19	6.92g1h1	1.503h-j	0.762u-x	0.222c1-e1	0.957c1d1	6.55f1g1	2.51a1-c1	9.06g1h1	2.963f-l	48.48y	8.41o
S3	G19	5.58k1l1	0.527za1	0.923m-p	0.371w-z	1.519b	6.62f1g1	2.64a1b1	9.27g1	2.08e1	56.65o	6.49z
S1	G20	10.3q-t	2.244b	0.482g1h1	0.118i1j1	1.004a1b1	11.94f	5.06j-l	17.01ij	3.153b-f	41.18j1	7.78u
S2	G20	9.37v-x	1.527hi	0.743v-y	0.196d1-f1	1.05xy	8.42vw	4.07qr	12.49v-x	3.011e-j	41.58i1	5.53b1
S3	G20	6.61h1i1	1.321no	0.901pq	0.328a1	1.326hi	8.88tu	3.43uv	12.31w-y	2.448u-b1	52.01t	10.69a
S1	G21	8.1a1-d1	1.606g	0.464h1	0.096j1k1	0.796j1-l1	8.15wx	2.39b1-d1	10.54d1e1	2.681o-t	35.19p1	7.84tu
S2	G21	6.56h1-j1	0.559zya1	0.722x-a1	0.188e1-g1	1.118tu	6.19h1i1	2.31c1d1	8.51i1j1	2.99e-k	38.71l1	8.71j-l
S3	G21	4.88m1	1.077rs	0.872p-r	0.203d1-f1	1.516bc	6.04i1	2.17d1	8.21j1	2.179c1-e1	50.71uv	9.23g
S1	G22	12.74e-g	1.029st	0.457h1	0.056k1	0.809i1-k1	11.43h-j	4.72mn	16.15l	2.873i-o	47.19a1	3.69i1j1
S2	G22	10.75m-q	2.021cd	0.684z-b1	0.192d1-g1	1.327hi	7.95x-a1	3.24v-y	11.21b1	2.811j-p	50.85u	7.98r-t
S3	G22	7.38e1g1	1.467k	0.882pq	0.195d1-f1	1.531b	8.11xy	3.08xy	11.19b1	2.571q-y	63.36h	7.22wx

### Principal component analysis (PCA)

**Fig. 1:**
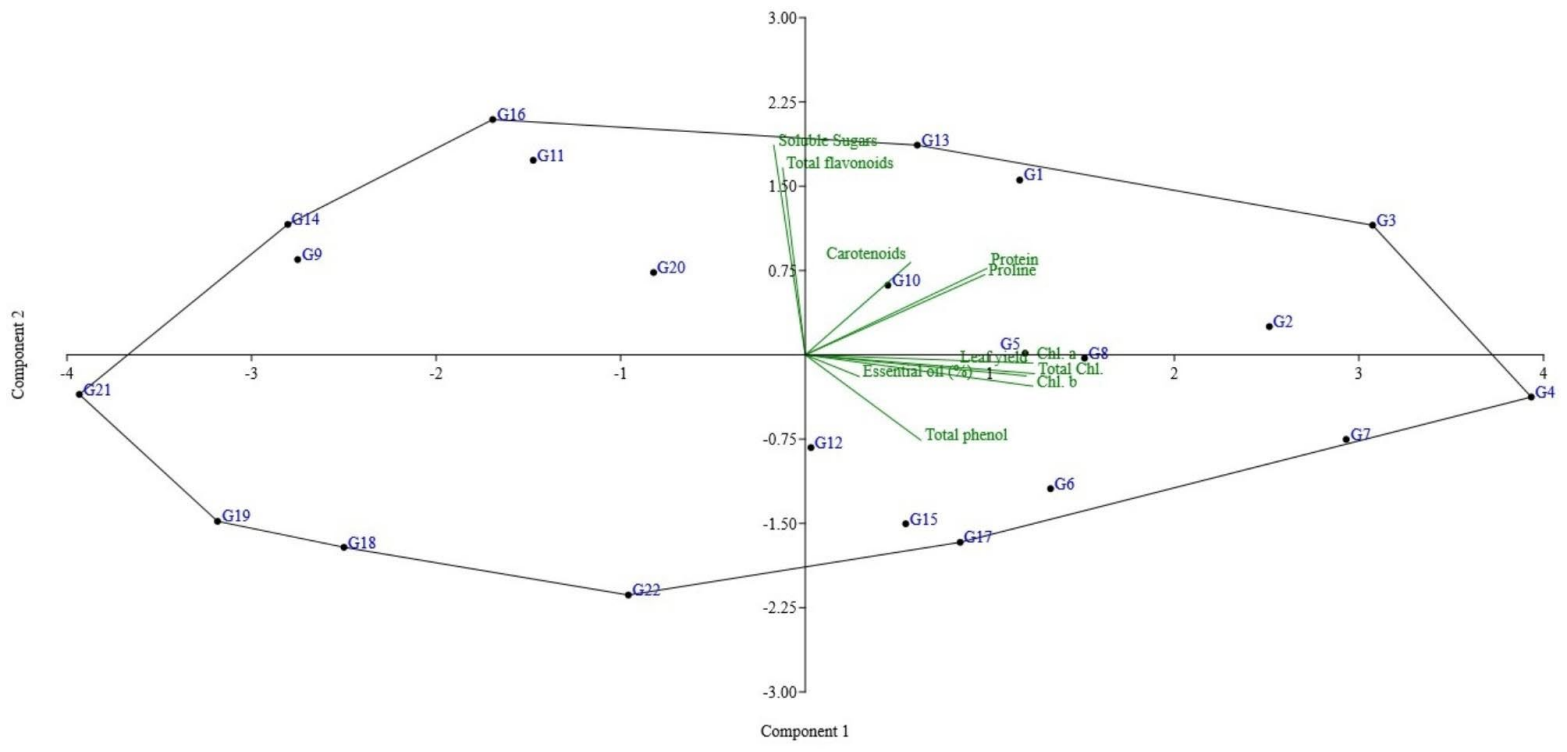
PCA scatter plot across 22 accessions of basil under normal conditions

**Fig. 2:**
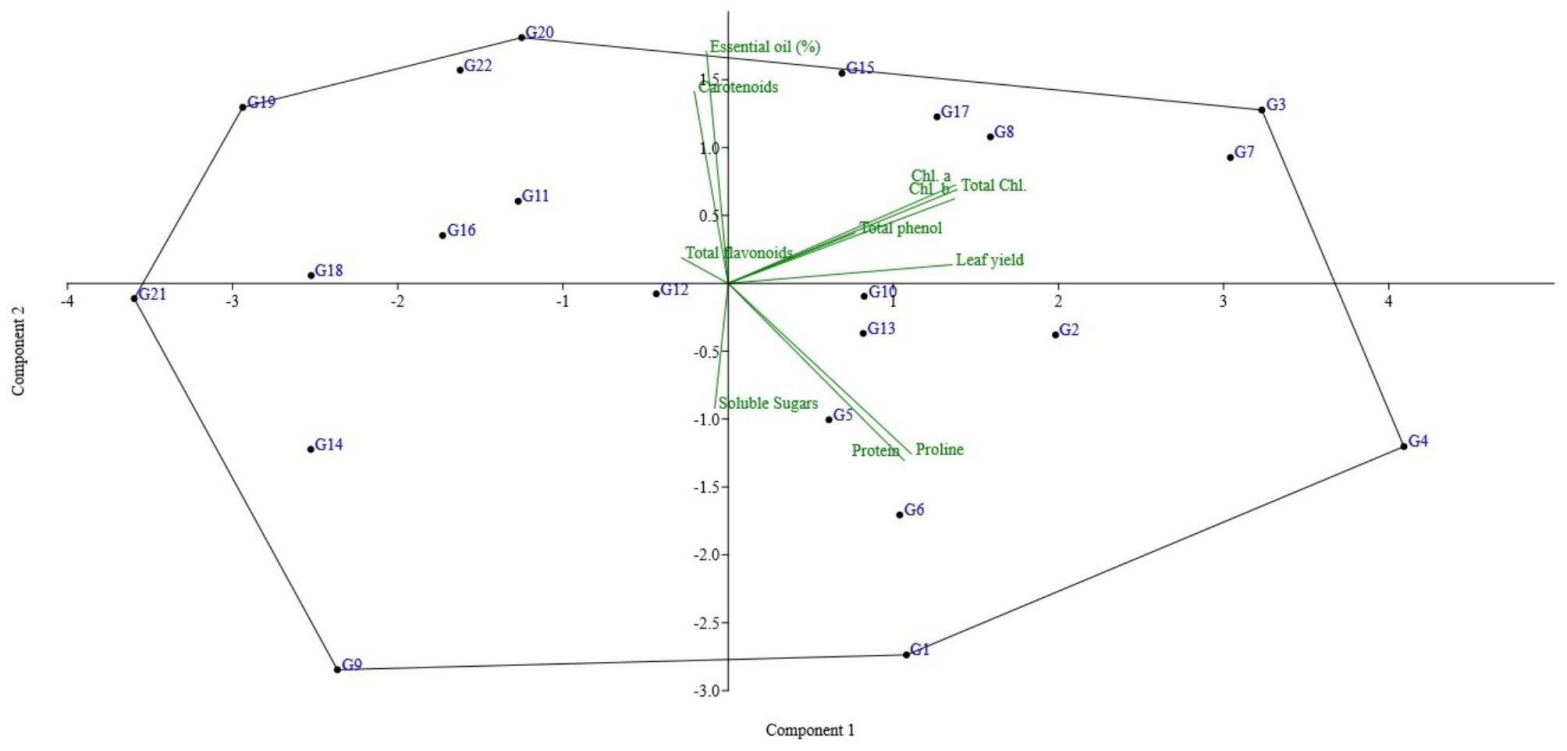
PCA scatter plot across 22 accessions of basil under moderate drought conditions

**Fig. 3:**
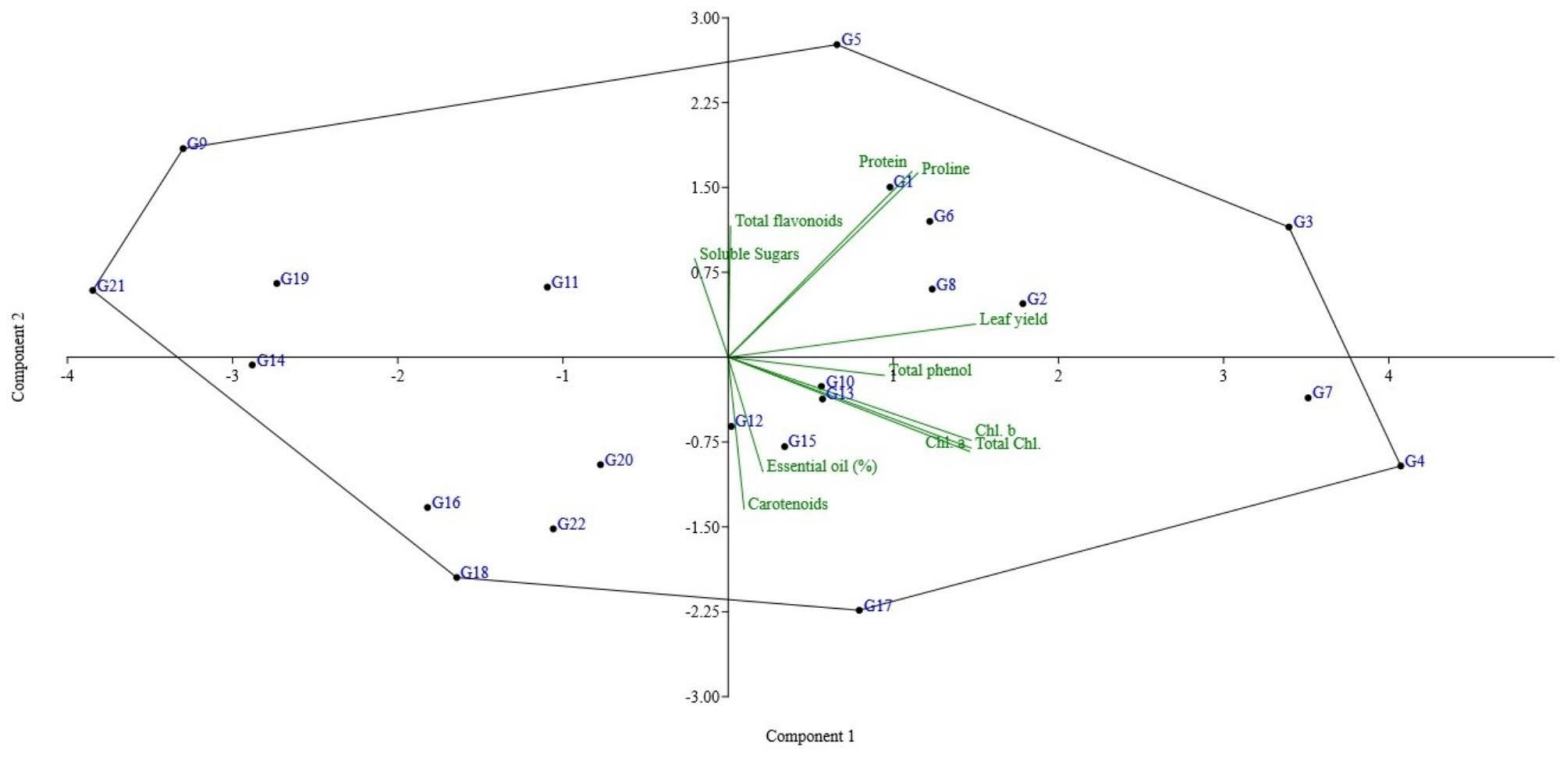
PCA scatter plot across 22 accessions of basil under severe drought conditions

The percentage of total variation explained by various principal component groups and their relationship to the investigated traits are displayed in the rotating component matrix. The PCA in normal conditions (Fig. 1), moderate drought (Fig. 2), and severe stress conditions (Fig. 3) was used to identify tolerant accessions. The results of PCA in normal conditions (Fig. 1) showed that the first two components explained about 59.51% of the variation (PC1 = 44.22% and PC2 = 15.29%), and the important and influential traits in the first component included leaf yield, protein, proline, chlorophyll a, chlorophyll b, total chlorophyll, and total phenol. The second PC is mostly contributed by soluble sugars and total flavonoids, and PC3 is contributed by essential oil. In the first component, traits that were related to yield and drought tolerance, such as protein, proline and sugar, had a positive and increasing effect. So, the accessions located in the first region of the biplot (G1, G2, G3, G5, G10, and G13) can be identified as tolerant accessions (Fig. 1).

The PCA-biplot in moderate drought showed that PC1 exhibited about 42.35% of the total variability and was explained principally by leaf yield, proline, chlorophyll a, chlorophyll b, total chlorophyll, and total phenol (Fig. 2). The second PC accounted for about 16.55% of the total variation and was mostly contributed by essential oils and carotenoids. The PC3 explained about 11.85% of the total variability and is contributed by soluble sugars and total flavonoids. The accessions located in the first region of the biplot (G3, G7, G8, G17, and G15) can be identified as tolerant accessions (Fig. 2) due to the positive effect of yield and drought tolerant such as protein, proline, and sugar in this region.

Under extreme drought conditions, the PCA revealed four principal component groups with an eigenvalue of higher than one, contributing 50.59% variability (Fig. 3). The PC1, PC2, PC3, and PC4 groups each provided a variable amount of 43.21, 14.64, 12.67, and 10.07%, respectively. Different criteria affected PC groups differently, both positively and negatively. In the PC1, leaf yield (0.428), protein (0.318), proline (0.328), chlorophyll a (0.418), chlorophyll b (0.420), total chlorophyll (0.420), and total phenol (0.270) recorded the highest variability. Maximum variability was observed for protein (0.469), proline (0.465), carotenoids (−0.384), total flavonoids (0.331), and essential oil (−0.288) in the PC2 group, whereas the essential oil (0.574), soluble sugars (0.516), total phenol (0.462), and total flavonoids (0.381) recorded higher variability than the other traits in the PC3 group. Henceforth, it came to light that the PC1 group exhibited the utmost diversity (amounting to 43.21%) when it comes to traits that contribute to drought tolerance, surpassing all other groups in this regard. Amidst the water scarcity environment, the plant species were predominantly clustered towards positive values on both PC1 and PC2 dimensions. Notably, G1, G2, G3, G5, G6, and G8 genotypes presented a profound inclination towards attributes such as leaf yield, protein content, proline concentration,total flavonoids level, and soluble sugars abundance (Fig. 3). Consequently,the identification of these accessions that exhibit exceptional performance in relation to these particular traits allows them be recognized as possessing an admirable capability to withstand and endure periods of drought stress.

### Cluster analysis

**Fig. 4:**
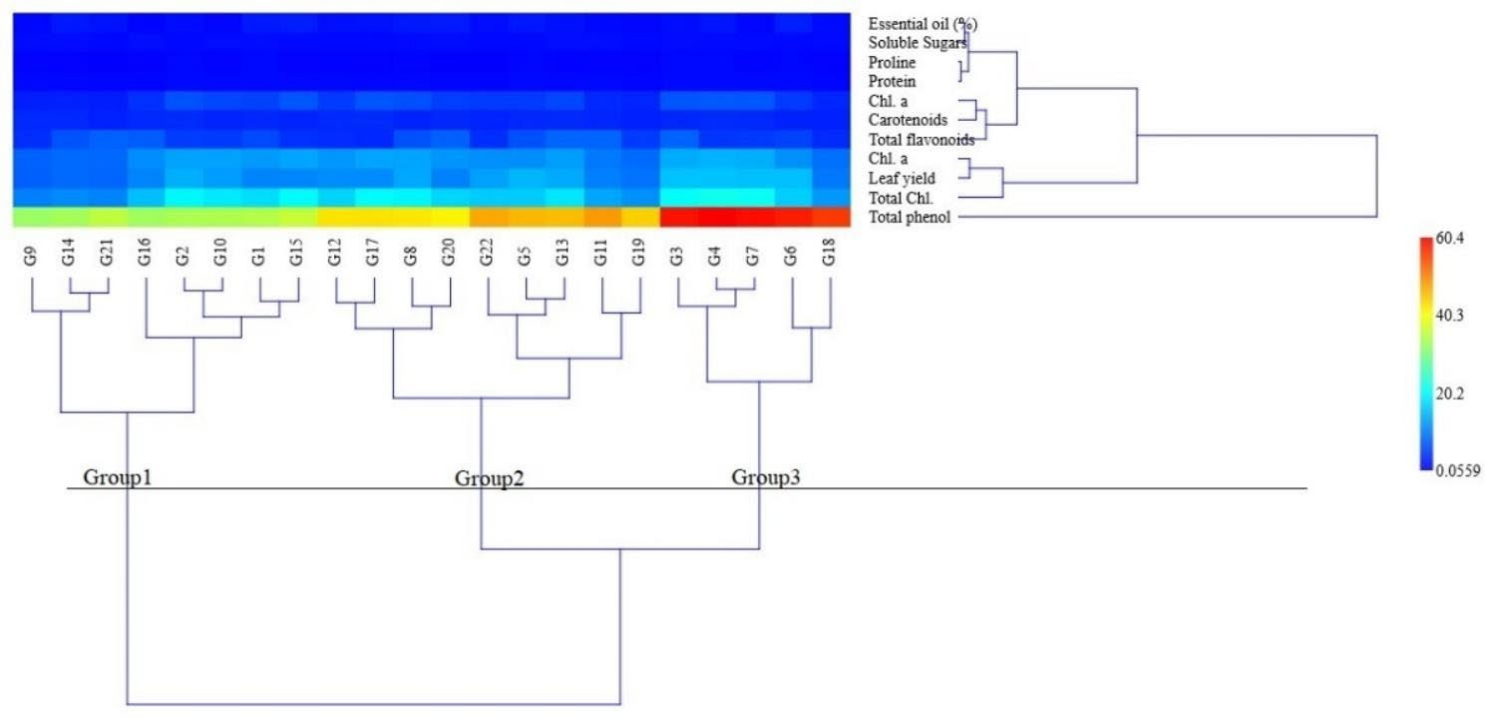
Ward cluster analysis of basil accessions under normal conditions

The accessions were divided into three clusters under normal conditions based on the examined attributes by Ward method cluster analysis, suggesting the presence of more genetic diversity among the accessions in distinct clusters, as shown in Fig. 4. Group 1 included G1, G2, G9, G10, G14, G15, G16, and G21 accessions. The accessions G5, G8, G11, G12, G13, G17, G19, G20, and G22 were placed in the second group, and accessions G3, G4, G6, G7, and G18 were included in the third group. The average traits of the third group were higher than the total average for most of the traits, so it was identified as a tolerant group. In addition, the average traits of the first group were lower than the total average identified as a sensitive group. Finally, the second group was identified as the semi-tolerant group.

**Fig. 5:**
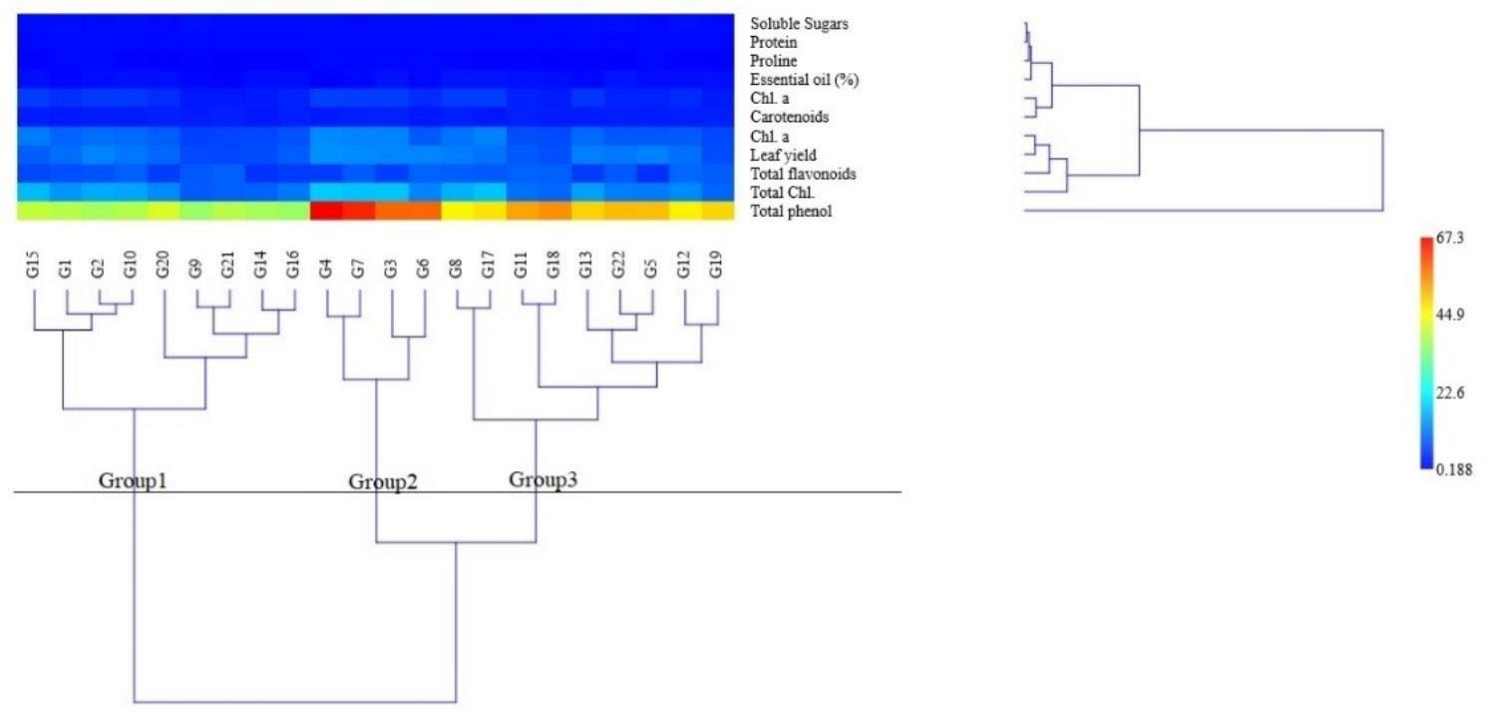
Ward cluster analysis of basil accessions under moderate drought conditions

The basil accessions were placed in three groups under moderate drought conditions (Fig. 5) using the Ward method with the cophenetic correlation coefficient equal to 73.14%. The average traits of the second group (G3, G4, G6, and G7) were higher than the average of all groups for most of the traits, so it was identified as a tolerant group. In addition, the average traits of the first group (G1, G2, G9, G10, G14, G15, G16, G20, and G21) were lower than the average of all groups for most of the traits, so it was identified as a sensitive group. Finally, the average traits of the third group (G5, G8, G11, G12, G13, G17, G18, G19, and G22) were in the middle of the other two groups, and the accessions of this group had been identified as semi-tolerant.

**Fig. 6:**
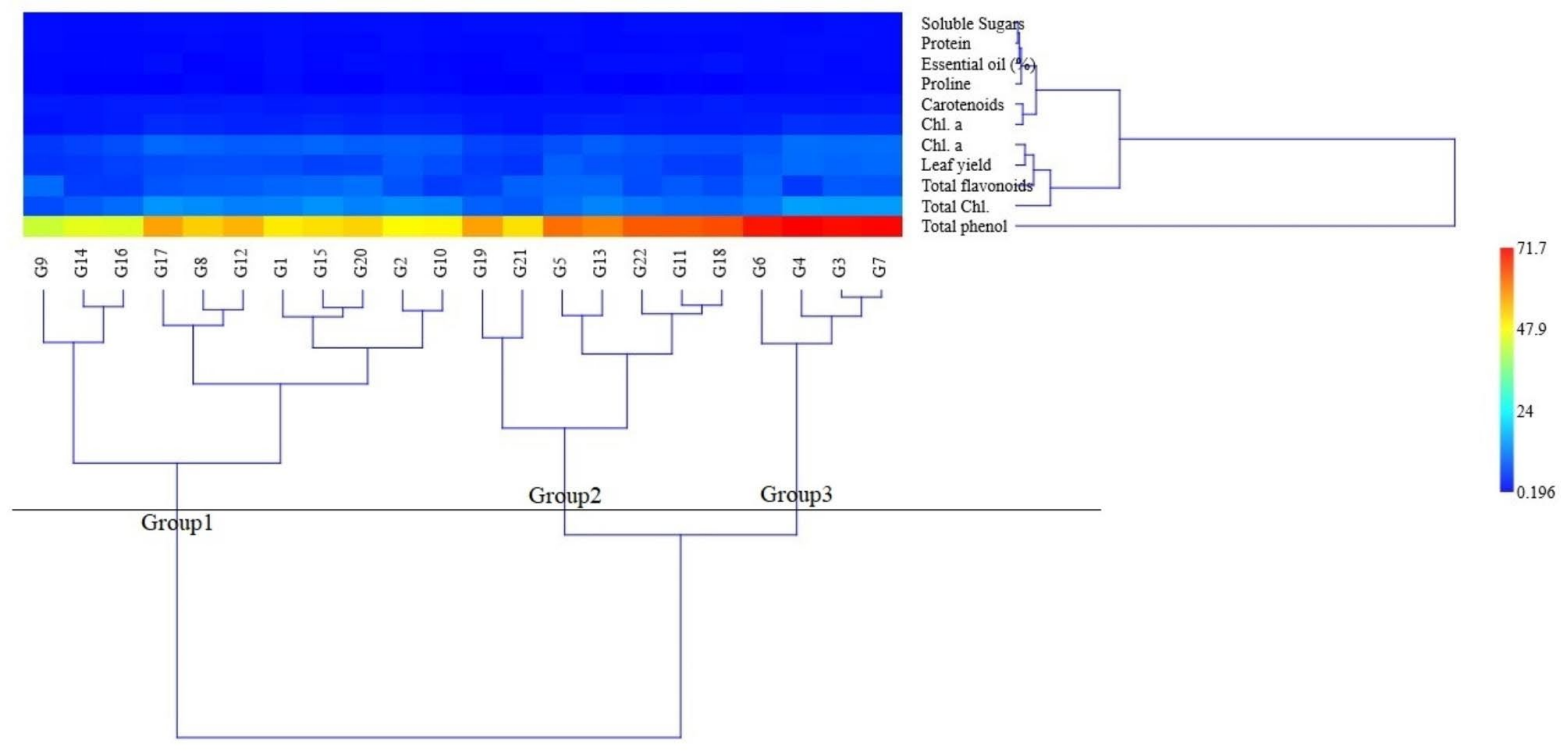
The ward cluster analysis of basil accessions under severe drought conditions

The accessions of basil had been split into three groups by cluster analysis under severe drought conditions using the Ward method (Fig. 6), with a cophenetic correlation coefficient equal to 69.1%. The average of the traits of the third group (G3, G4, G6, and G7) was higher than the average of all groups for most of the traits, so it had been identified as a tolerant group. In addition, the average of the traits of the first group (G1, G2, G8, G9, G10, G12, G14, G15, G16, G17, and G20) was lower than the average of all groups for most of the traits, so it had been identified as a sensitive group. The average traits of the second group (G5, G11, G13, G18, G19, G21, and G22) were in the middle of the other two groups, and the accessions of this group had been identified as semi-tolerant. The outcomes of this approach agreed with those of the mean comparison and PCA. The accessions of the tolerant group in this study performed better than other accessions in terms of yield, essential oil, proline content, and total protein (Table 5).

## Discussion

The development of basil drought-tolerant varieties is one of the most important breeding objectives. Screening of diverse basil accessions under limited water conditions is an efficient breeding programs [38, 39]. In the current study, basil accessions showed high variation under normal and water stress conditions. Drought stress is the main cause of yield reduction. To reduce the possibility of a global food crisis in the future, it is necessary to develop methods to identify and select drought-tolerant plants. The degree of drought tolerance of the species and the mechanisms involved in plant survival in arid and semi-arid environments must be established for this purpose [40]. Plant variety plays a significant role in determining tolerant genotypes in biotic and abiotic stress conditions [41].

Leaf yield is one of the important traits of basil because it is used as a vegetable. Drought stress reduced leaf yield in basil plants. The leaf yield reduction was between − 6.55% and 25.33%, and − 32.01% and − 42.97% for moderate and severe stress conditions, respectively. Accessions that showed the lowest amount of reduction can be identified as tolerant. But the yield value should also be considered. Crossing tolerant with high-yielding genotypes and examining their progeny is one of the ways to improve the identification of high-yielding as well as tolerant genotypes.

A trait that can be impacted by genotype, growing circumstances, growth stage, etc. is essential oil content [42, 43]. In this study, both the drought and the cultivar, as well as how they interacted, had an impact on the essential oil content. The Lettuce accession has the highest essential oils, followed by Horapa and Black. In some genotypes, the percentage of essential oil increased with drought level, but in others it decreased, like the results of some other research [22–24]. According to results from other basil studies [39, 44, 45] and other species [46–48], water stress can have either a good or detrimental impact on the content of essential oils. The cultivars showed a wide range of essential oils. The use of diverse genetic material and varying environmental conditions may account for differences in basil essential oil content between this study and previous ones. The quantity and quality of essential oils might alter depending on genetic and environmental factors. Other studies have revealed significant changes in essential oil components’ qualitative and quantitative composition as a result of unfavourable conditions [22, 24].

The protein results showed that the amount of protein increased in tolerant cultivars. Abiotic stresses usually cause the incomplete functioning of proteins [49]. Proteins that increase in plants during stress may be a form of nitrogen storage that is later used by the plant or may play a role in osmotic adaptation. Moreover, they may cause their reuse for the synthesis of smoothie-like proteins or structural proteins or change the structure of the cell wall. These proteins may be synthesised in response to stresses or may be structurally present in small concentrations [50].

The results of proline content showed that the content increased linearly with the increase in stress (Table 5). The highest increase in proline among accessions belonged to Italian Genovese, Particolored, and Red Rubinunder, respectively, in severe and moderate stress conditions. The higher accumulation of proline in basil plants under drought stress was an adaptation for drought tolerance, which in turn helped the plant to survive and reproduce under drought conditions. Although it has been shown that proline accumulation is linearly related to increasing drought stress, it is not always true to say that genotypes with higher proline content are always drought-tolerant. The other studies also showed a significant increase in the amount of proline [51, 52]. The results of many papers on other plants also show an increase in proline in improving tolerance to drought stress [53–55]. Proline accumulation is a widespread physiological response in many plants in response to a wide range of biotic and abiotic stresses [56, 57]. There is a lot of information about the accumulation of proline as a common physiological response in many plants under different environmental stress conditions [58, 59]. The role of proline in increasing stress tolerance has been reported in many studies, but there are conflicting reports regarding its use as a tolerance index [60]. Plants have different strategies, including biochemical mechanisms, to reduce the effects of stress. For example, proline can act as a compatible solvent, an osmotic protector, and a protector for cytosolic enzymes and cell organelles. In addition, proline can be used as a carbon and nitrogen source, membrane stabiliser, and accelerator for free radicals [57].

The total phenol and flavonoids increased with drought levels. The highest phenol was observed in Lemon, Purple and Red Rubin accessions, respectively. Moreover, the highest flavonoids belonged to Black, Cinnamon and Midnight accessions, respectively. Other studies also showed an increase in these traits in basil under drought stress conditions [61, 62]. Moreover, various reports in different plants have shown that under stress conditions, the increase of these indices can help with stress tolerance, and based on that, tolerant accessions can be identified [63, 64]. Also, in the study of Varela, Arslan [65], phenolic compounds have been used as an indicator to identify stress-tolerant accessions. Phenols are powerful antioxidant compounds in plant tissues under dry conditions. Due to their skeletal structure, these compounds will play an important role in eliminating the oxygen-free radicals produced in stressful conditions [66, 67].

The results showed the reduction of chlorophyll a, b, total chlorophyll, and carotenoids with drought levels. However, this decrease was not the same in all accessions. The results of other studies also showed a reduction of these traits under drought stress conditions [15, 39, 44, 68]. The chlorophyll decrease can be due to the change in nitrogen metabolism towards the production of compounds such as proline, which is used in osmotic regulation [69]. The soluble sugar results increased with drought levels. Some accessions showed an increase in soluble sugars and some showed a decrease in the drought level, and it was in line with other studies [44, 51] on basil. Researchers have reported an increase in soluble sugars in plants under stress conditions. They stated that the reason is due to the breakdown of insoluble carbohydrates, the synthesis of osmotic substances from non-photosynthetic pathways, growth arrest, a reduction in the rate of transfer of substances, and the increase in sucrose synthesis (due to the activation of the sucrose phosphorosynthase enzyme) [70]. Research showed an increase in soluble sugars. The accumulation of soluble sugars helps to regulate the osmolarity in plant cells, preserve biomolecules and membranes, store carbon materials, and neutralise free radicals [59]. More tolerant accessions have better osmotic regulation by accumulating and maintaining more soluble sugars. These conditions make the turgor pressure necessary for growth in stress conditions better maintained [71].

To further clarify the association between more than two traits at once, multivariate analysis is also used, such as cluster analysis and PCA. The extensive range of varieties displayed by cluster analysis helps in the identification of tolerant accessions. An interpolated biplot between PC1 and PC2 revealed a distinct pattern of genotype clustering along the vector line. Plotted closer to the vector line were specific accessions that performed exceptionally well for a given characteristic. By comparing the PCA method under normal and severe stress conditions, as well as the effective traits of the components, the first part of the biplot was identified as the drought-tolerant area. The accessions located in this area are tolerant, and G3, G4, G6, G7, and G18 were identified as tolerant accessions. Due to differences in how well 20 basil accessions tolerated drought-induced stress, hierarchical cluster analysis and PCA identified contrasting variances in the accessions and grouped the accessions into discrete clusters. Additionally, cluster analysis was used to classify the accessions according to their characteristics under drought conditions and to identify the group that displayed the highest level of tolerance. Cluster analysis in all three conditions classified the accessions into three categories, and these groupings were very similar. G3, G4, G6, and G7 were placed in one group in each condition. The average traits of this group were higher than those of other groups. The results of PCA largely confirmed the results of cluster analysis. Their high dissimilarity is due to the method of PCA not using all information about the traits. In the present study, using multivariate statistical methods, basil cultivars were grouped, and superior and weaker genotypes were identified in each environment. Moreover, the important and key variables in differentiating and justifying the total variation were determined using PCA. In total, G3, G4, G6, and G7 accessions were in the top group in all conditions and were identified as tolerant genotypes.

## Conclusion

This study showed the superior ability of physiological and biochemical parameters and provided evidence to determine which crop is most tolerant to water stress. Moreover, this was the main criterion for examining a large number of lines and varieties and determining their response to this dangerous environmental factor. In this regard, hierarchical clustering and PCA biplot analysis had played a fruitful role in screening all basil accessions in terms of their response to water stress. This point is considered a essential statistical factor in saving time when considering the evaluation of these accessions, again in other environments characterised by a scarcity of water resources. According to the study’s findings, the accessions Red Rubin, Lemon, Lettuce, and Purple can be suggested as the most drought-tolerant accessions and can also be utilised as donors for further development of drought-tolerant varieties. This research contributes to the genetic enhancement of basil for drought tolerance.

## Data Availability

The data used to support the findings of this study are included within the article.
